# Supplementary material for: Processed Animal Proteins from Insect and Poultry By-Products in a Fish Meal-Free Diet for Rainbow Trout: Impact on Intestinal Microbiota and Inflammatory Markers
Source: Int J Mol Sci. 2021 May 21;22(11):5454. doi: 10.3390/ijms22115454 (PMC8196822; doi:10.3390/ijms22115454)
Supplement: Supplementary file 1 [file ijms-22-05454-s001.zip › Table S1.pdf]

	Experimental diets							
**Ingredients (g/100g)**	**CF**	**CV**	**H10**	**H30**	**H60**	**P30**	**P60**	**H10P50**
Fishmeal[1]	47.5	-	-	-	-	-	-	-
CPSP90[2]	5.0	5.0	5.0	5.0	5.0	5.0	5.0	5.0
SBM	-	23.0	20.4	16.0	9.0	16.0	9.0	9.0
Protein-rich veg. mix[3]	3.8	34.9	30.4	21.9	10.2	21.2	8.0	8.3
Hermetia meal[4]	-	-	7.8	22.7	45.0	-	-	7.8
P[5]	-	-	-	-	-	17.8	36.0	29.7
Whole wheat + Pea meal	22.6	7.1	9.2	9.6	9.2	16.8	21.6	18.0
Fish oil	15.1	4.4	4.4	4.4	4.4	4.4	4.4	4.4
Vegetable oil mix[6]	4.3	17.7	16.7	14.8	12.0	15.5	13.4	13.2
Vit & Min Premix	1.7	1.7	1.7	1.7	1.7	1.7	1.7	1.7
Dicalcium Phosphate	-	3.0	3.0	2.8	2.7	0.6	-	1.8
Betaine HCl	-	1.5	-	-	-	-	-	-
L-Lysine	-	1.2	0.9	0.7	0.5	0.6	0.6	0.8
DL-Methionine	-	0.45	0.45	0.40	0.35	0.35	0.25	0.25
L-Tryptophan	-	0.05	0.02	-	-	0.04	0.05	0.03
**Proximate composition**								
Dry Matter	92.4	91.2	90.5	91.2	91.1	90.7	94.0	92.9
Crude protein	42.0	42.1	41.9	41.5	42.0	41.8	42.2	41.9
Crude lipids	23.9	23.9	24.2	23.8	24.1	23.9	24.0	24.2
Ash	9.5	8.0	8.2	8.3	8.6	6.7	6.8	8.4
Chitin#	-	-	0.37	1.1	2.1	-	-	0.37
Gross Energy (MJ/kg)	22.4	21.9	22.5	21.9	22.5	22.5	22.9	22.9
