# Supplementary material for: Processed Animal Proteins from Insect and Poultry By-Products in a Fish Meal-Free Diet for Rainbow Trout: Impact on Intestinal Microbiota and Inflammatory Markers
Source: Int J Mol Sci. 2021 May 21;22(11):5454. doi: 10.3390/ijms22115454 (PMC8196822; doi:10.3390/ijms22115454)
Supplement: Supplementary file 1 [file ijms-22-05454-s001.zip › Table S2.pdf]

[A]

**Phylum**	**CV vs CF**	**CV vs H10**	**CV vs H30**	**CV vs H60**	**CV vs P60**	**CV vs H10P50**	**CF vs H10**	**CF vs H30**	**CF vs H60**	**CF vs P30**	**CF vs P60**	**CF vs H10P50**	**H10 vs P30**	**H10 vs P60**	**H30 vs P30**	**H30 vs P60**	**H60 vs P30**	**H60 vs P60**	**P30 vs H10P50**	**P60 vs H10P50**
Actinobacteria																		0.037		
Bacteroidetes																	0.016			
Fusobacteria	0.014						0.002	0.015	0.012	0.016	0.013	0.035								
**Class**																				
Erysipelotrichi			0.026	0.041																
Fusobacteriia							0.012													
Gammaproteobacteria				0.041																
**Order**																				
Actinomycetales														0.05		0.041				
Bacteroidales																	0.026			
Erysipelotrichales		0.036	0.010	0.028											0.023	0.029	0.05			
Fusobacteriales							0.005	0.040		0.038	0.032									
Pseudomonadales				0.028																
**Family**																				
Actinomycetaceae		0.012	0.019	0.035		0.042							0.011	0.003	0.019	0.010	0.05	0.023	0.041	0.011
Corynebacteriaceae													0.049							
Bacillaceae														0.05						
Paenibacillaceae				0.042																
Clostridiaceae				0.042																
Eubacteriaceae				0.027													0.031	0.023		
Erysipelotrichaceae		0.047		0.035											0.027	0.034				
Fusobacteriaceae							0.002													

[B]

Genus	CV vs CF	CV vs H10	CV vs H30	CV vs H60	CV vs P60	CV vs H10P50	CF vs H10	CF vs H30	CF vs H60	CF vs P30	CF vs P60	CF vs H10P50	H10 vs P30	H10 vs P60	H30 vs P30	H30 vs P60	H60 vs P30	H60 vs P60	P30 vs H10P50	P60 vs H10P50
Actinomyces		0.019	0.029	0.041		0.040							0.017	0.006	0.022	0.013	0.05	0.027		0.014
Oceanobacillus			0.034	0.029									0.044	0.035	0.015	0.013	0.015	0.013		
Bacillus				0.036																
Pseudoramibacter_Eubacterium					0.029												0.039	0.029		
Dorea		0.019	0.029	0.029		0.017								0.020		0.027		0.027		0.014
Paeniclostridium						0.044														
Erysipelothrix			0.015	0.029											0.015	0.019	0.039	0.048		
Enterococcus			0.027	0.027									0.034		0.016	0.022	0.021	0.051		
Cetobacterium							0.031													
Pseudomonas				0.041																
