# Supplementary material for: Processed Animal Proteins from Insect and Poultry By-Products in a Fish Meal-Free Diet for Rainbow Trout: Impact on Intestinal Microbiota and Inflammatory Markers
Source: Int J Mol Sci. 2021 May 21;22(11):5454. doi: 10.3390/ijms22115454 (PMC8196822; doi:10.3390/ijms22115454)

Figure S1. Spearman's correlation between relative abundance of gut microbial genera and the expression of selected markers in midgut biopsies. A positive correlation is indicated by dark red, a negative correlation by dark blue. Stars indicate statistically significance after FDR correction (*$p < 0.05$, **$p < 0.01$, ***$p < 0.001$). Families and genera were reported as "Unassigned" when they could not be assigned to any genus (g) or family (f) within the reference database (http://greengenes.lbl.gov), at a percent-age sequence homology of 95% or 90%, respectively for genus and family.

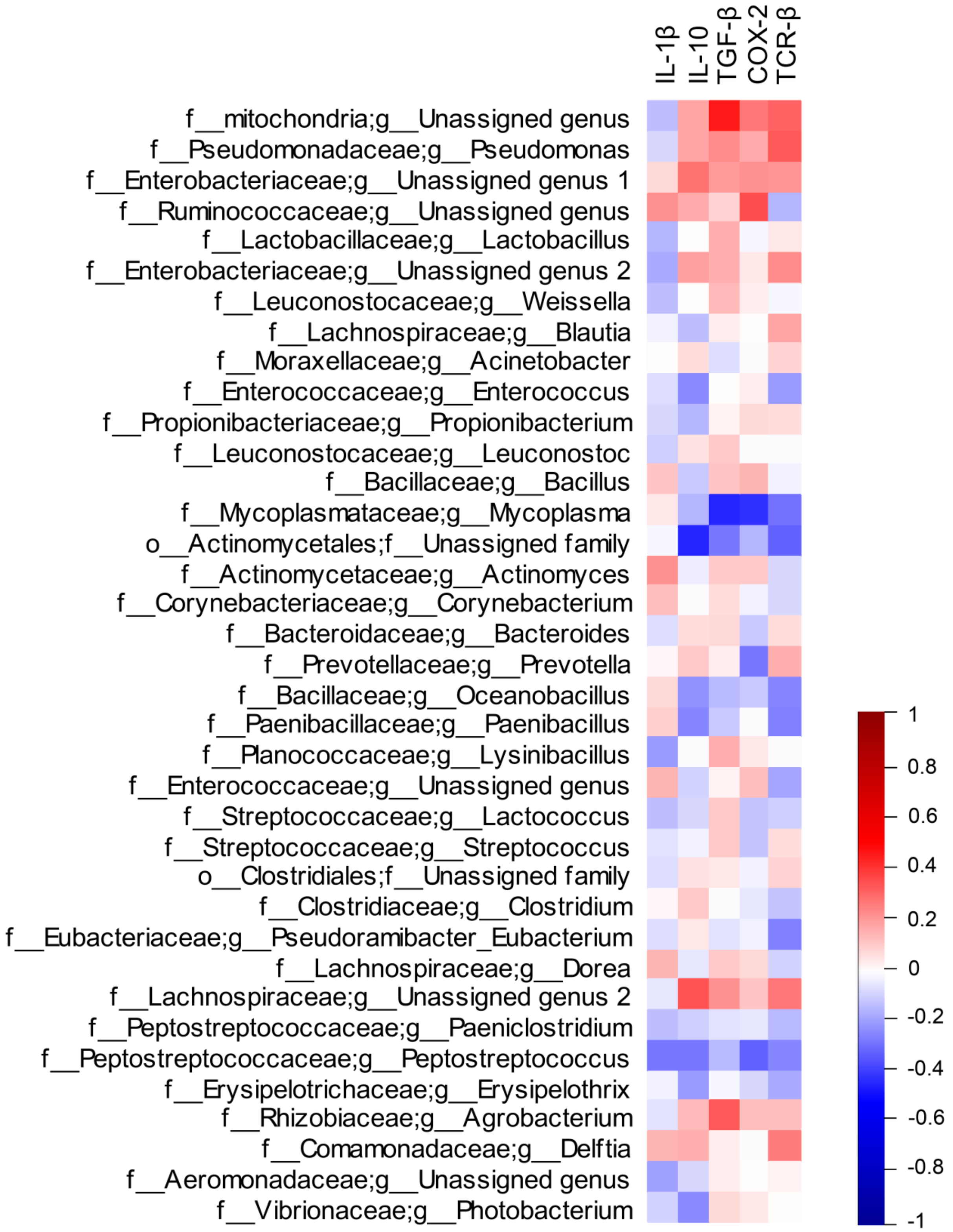

Supplement: Supplementary file 1 [file ijms-22-05454-s001.zip › Figure S1_revision.pdf]
